# TTC36 inactivation induce malignant properties via Wnt-β-catenin pathway in gastric carcinoma

**DOI:** 10.7150/jca.47292

**Published:** 2021-03-05

**Authors:** Lei Song, Xiaonong Guo, Fei Zhao, Wei Wang, Zhifang Zhao, Long Jin, Chengli Wu, Jibin Yao, Zhongren Ma

**Affiliations:** 1 Key Laboratory of Biotechnology and Bioengineering of State Ethnic Affairs Commission, Biomedical Research Center, Northwest Minzu University, Lanzhou 730030, Gansu, China.; 2 Department of Medicine, Northwest Minzu University, Lanzhou 730030, Gansu, China.; 3 Department of Urology, Institute of Urology, Gansu Nephro-Urological Clinical Center, Key Laboratory of Urological Diseases in Gansu Province, Lanzhou University Second Hospital, Lanzhou 730030, Gansu, China.; 4 Department of Surgical Oncology, Gansu Provincial Hospital, Lanzhou 730000, Gansu, China.

**Keywords:** gastric carcinoma, Hsp70 binding protein 21 (HBP21), beta-catenin, apoptosis, proliferation, xenograft

## Abstract

**Objective:** Tetratricopeptide repeat (TRP)-mediated cofactor proteins are involved in a wide range of cancers. TTC36 is little studied member of TRP subfamily. This study aimed to investigate the role of TTC36 in human gastric carcinoma (GC) and explore the potential underlying mechanisms.

**Methods:** The analysis of TTC36 differential expression in GC was conducted using data from TCGA and a human tissue microarray. And effects of TTC36 expression on the prognosis of patients with gastric carcinoma were analyzed using the data from the GEO database. Lentivirus was transfected into the cell lines of AGS and BGC823 to construct overexpression and knocked down TTC36 cell model respectively. The effect of TTC36 expression on the growth, apoptosis and cell cycle of cells was explored *in vitro*. Downstream molecules were detected by western blotting. GSEA predicted signal pathway and related proteins were then detected.

**Results:** TTC36 expression in human GC tissues was found significantly lower than that in adjacent normal tissues and closely related to clinical prognosis. The overexpression of TTC36 notably inhibited tumor progression, cell cycle G1/S transfer and increased apoptosis in AGS cells. Conversely, the opposite effects were observed when TTC36 was suppressed in BGC823 cells. The expression of cleaved caspase3, Survivin, cyclin D1 and c-Myc were consistent with the phenotype in TTC36 operated GC cell lines. Intriguingly, GSEA analysis predicted Wnt-β-catenin pathway involved in TTC36 induced effects in GC cells, expression of β-catenin and downstream molecules such as TCF4, c-jun and pAKT were found negative related to TTC36 expression in GC cells. Notably, treatment with the Wnt/β-catenin inhibitor XAV939 dramatically attenuated the effects of TTC36 in GC cells.

**Conclusion:** These results signify a critical role for TTC36 as a tumor suppressor in gastric carcinoma via regulating Wnt-β-catenin pathway.

## Introduction

Gastric carcinoma is the fifth most common type of cancer, and is the third leading cause of cancer-related death in the world 1. The main treatment regimens for GC include endoscopic resection, partial or total gastrectomy and peri-operative chemotherapy. Despite advances in conventional treatment and surgical interventions, a high percentage of GC patients still have poor survival 2, 3. So improvements in long-term survival depend on the availability of newer drugs or better understanding about on GC 4. To find novel biomarkers that can contribute to predicting prognosis and developing effective therapeutic strategies against GC is necessary.

Tetratricopeptide repeat domain 36 (TTC36) has rarely been reported in the past several decades, which was also known as HBP21 (Hsp70 binding protein 21) encodes a protein composed of three adjacent tetratricopeptide repeat (TRP) motifs 5. Each of these TPR motifs possesses a characteristic 34-amino acid sequence, which folds to shape a helix-turn-helix motif 6. The adjacent motifs present in a parallel manner and form a right-handed superhelix, whose groove structure can accommodate the complementary domain of various ligands and further mediate protein-protein interactions 7. This structure can act as scaffolds for interaction with the C-terminal domain of Hsp70, involved in a variety of cellular functions that require protein-protein complexes formation in numerous cellular functions such as chaperone, cell-cycle, transcription, protein transport and degradation 8. Xie *et al.* reported TTC36 as a molecular chaperone exert important physiological significance of HPD regulation by TTC36-STK33-PELI1 complex in tyrosinemia related neurological disorders 9. The roles of some TPR proteins in human tumors have been well studied, for instance, among TPR cochaperones, Hip has been identified as anti-apoptotic protein in leukemia cells 10, but in another study, DNAJC25 has been reported as a proapoptotic gene in hepatocellular carcinoma 11. So far, the function of TTC36 in human tumor has not been studied extensively. Previously, TTC36 is found to promote apoptosis through bind with HSP70 and inhibit its interaction with Bax and subsequently promote the cytoplasm-to-mitochondria translocation of bax in hepatocellular carcinoma 12. So TTC36 has been demonstrated as a chaperone with proapopotic ability. However, the expression level of TTC36 and its biological role in GC remain unclear. We hypothesize whether it will also have tumor suppressing function in other neighboring digestive system tumors especially GC, and want to find it out.

This study aimed to explore the function and mechanisms of TTC36 in GC. The expression of TTC36 in GC was confirmed by public datasets and human tissue microarray. The obvious tumor suppressing function of TTC36 in GC cells was investigated *in vitro* and *in vivo*. In addition, the underlying mechanism of TTC36 on GC tumorigenesis was studied.

## Materials and methods

### Expression analysis by bioinformatic data mining

The data for mRNA expression (mRNA SeqV2) of human Gastric Carcinoma were retrieved from The Cancer Genome Atlas (TCGA) project (https://tcga-data.nci.nih.gov/tcga), TTC36 expression was extracted from TCGA RNA-seq data (STAD, Stomach adenocarcinoma) which contained 375 primary GC tissues and 32 surrounding non-cancer tissues, respectively. mRNA array data of GC gene profile in GSE66229 13 was downloaded from open Gene Expression Omnibus (GEO) database (https://www.ncbi.nlm.nih.gov/geo/). The differential-expressed mRNA data of the tumor tissues and normal tissues were then analyzed by "DESeq2" package using R software (version 3.5.1). The plots were generated by GraphPad Prism 7. The statistical significance in survival analysis was estimated according to the method of Kaplan-Meier using survifit (R package). *P* values were calculated by the log-rank test using survdiff (R package).

### Human tissue microarray

The tissue microarray (TMA) slide (cat. no. HStm-Ade080CD-01) was purchased from Shanghai Outdo Biotech Co., Ltd., Shanghai, China. Slide contained samples from 80 individual cases, with 6 cases of normal gastric mucosa, 5 cases of distal GC mucosa, 18 cases of mucosa adjacent to GC tissue, 23 cases of primary GC tissue, 10 cases of liver metastasis tissue, 5 cases of non-invasive lymph node and 13 cases of invasive lymph node.

### Validation of TTC36 expression on TMA by immunohistochemistry (IHC)

The TMA slide was deparaffinized in xylene for 15 min, rehydrated in graded solutions of ethanol, heated at 95 °C for 5 min in 10 mM sodium citrate buffer (pH 6.0) in a microwave oven for antigen retrieval. Endogenous peroxidase was inactivated by incubation with 3% H_2_O_2_ for 15 min. Non-specific staining was reduced by incubating the slide with normal goat serum (1:10; cat.no. C-0005; Jiangxi Haoran Bio-Pharma Co., Ltd., Shanghai, China) for 20 min at 37 °C. The optimal concentration of rabbit monoclonal antibody against TTC36 (Ab122507, Abcam) was determined by serial dilutions. The slide was incubated with primary TTC36 antibody (1:2000) at 37 °C for 60 min. Finally, the slide was stained with horseradish peroxidase-conjugated goat anti-rabbit IgG secondary antibody (1:1; cat.no.SP-9001; Zhongshan Biotechnology Co., Beijing, China) for 30 min at room temperature (RT). Reactions were carried out using the ultraView DAB detection kit. Slide were counterstained with haematoxylin, dehydrated, and mounted. The slide was then visualized using a bright field microscope under × 200 magnification. Immunohistochemistry was scored based on staining intensity and the percentage of positive cells. The immunoreactive score = staining intensity × percent of TTC-positive cells. Staining intensity was scored as 0, negative; 1, weak; 2, moderate; and 3, high. The percentage of positive-stained cells was scored as follows: 0, < 5%; 1, 5-25%; 2, 26-50%; 3, 51-75%; and 4, > 75%. Immunohistochemistry score of TTC36 was estimated in a blinded study by two independent observers.

### Gene set enrichment analysis (GSEA)

We used Gene set enrichment analysis (GSEA v2.2.3, http://www.broad.mit.edu/gsea/) to analyze the association between expression of TTC36 and signaling pathway. GSEA was carried out with the mRNA expression data from TCGA dataset. The TTC36 expression levels were divided into high expression group (with expression values above median) and low expression group (with expression values below median). Pre-defined gene sets in C2 collection (curated gene sets) from the Molecular Signatures Database-MsigDB (http://software.broadinstitute.org/gsea/msigdb/collections.jsp#H) were used for enrichment analysis. The number of permutations was set to 1,000. A gene set is considered significantly enriched when false discovery rate (FDR) < 0.25 and *P* value < 0.05.

### q-PCR analysis

Total cellular RNA was isolated using TRIzol reagent (Invitrogen, Carlsbad, USA) and quantified by spectrophotometry at 260nm using a NanoDrop ND-1000 (NanoDrop Technologies, Wilmington, DE, USA). cDNA synthesis was performed using M-MLV Reverse Transcriptase (K1622, Fermentas, USA). Quantitative real-time PCR was carried out using the SYBR Green PCR kit (#K0223, Thermo) on an ABI-7300 instrument (ABI, NY, USA). Real-time PCR was performed with the following conditions: predenaturate at 95 °C for 10 min followed by 40 cycles of denaturation at 95 °C for 15 s, annealing at 60 °C for 45 s and elongation at 60 °C for 1 min. The primers sequences were as follows: TTC36 (sense 5'-ATAAAGCCGTCGGGCCTCAC-3', antisense 5'-TCTGATGAGCACCGCAGTCC-3'), and GAPDH (sense 5'- AATCCCATCACCATCTTC-3', antisense 5'- AGGCTGTTGTCATACTTC-3'). Relative expression of TTC36 was calculated as 2^-ΔΔCt^ using GAPDH as an internal reference. All experiments were repeated in triplicate.

### Western blotting

The cell lysates were prepared by harvesting the cells in RIPA lysis buffer kit (BYL40825, JRDUN, Shanghai, China), and protein concentration were quantified using the Bradford assay (23223, Thermo, Waltham, MA, USA). Total protein (25 μg) was separated on 10% or 15% sodium dodecyl sulfate polyacrylamide gel electrophoresis (SDS-PAGE) and transferred to nitrocellulose membranes (Millipore; Merck KGaA). Membranes were blocked with 5% skim milk for 1 h at RT and incubated with rabbit monoclonal antibody against TTC36 (1:300) (Ab122507, Abcam), β-catenin (1:1000) (cat. 8480, Cell Signaling), survivin (1:1000) (cat.2808, Cell Signaling), CyclinD1(1:1000) (cat.2922, Cell Signaling), c-Myc (1:1000) (Ab32072, Abcam), cleaved caspase3 (1:500) (Ab2302, Abcam) and GAPDH (1:2000) (cat.5174, Cell signaling) overnight at 4 °C, and the membrane was rinsed 3 times in TBST. The membrane was then incubated with a goat anti-rabbit secondary antibody (1:1000) (A0208, Beyotime) for 1 h at RT followed by three washes with TBST. Finally, signals were visualized by enhanced chemiluminescence kit (WBKLS0100, Millipore) and scanned using the Electrophoresis Gel Imaging Analysis System (Tanon-5200).

### Cell culture

A normal human gastric mucosal epithelium cell line GES-1 and four GC cell lines AGS, BGC823, MGC-803 and MKN45 were purchased from the Cell Bank of Chinese Academy of Science (Shanghai, China). Cells were cultured in RPMI-1640 (Hyclone, SH30809.01B, USA), supplemented with 10% fetal bovine serum (GIBCO, 10099141, USA) and 1% double antibiotics (penicillin and streptomycin, Solarbio, P1400-100, China), incubated in a 5% CO_2_ incubator at 37 °C.

### Vector construction and lentivirus infection

Three siRNA against TTC36 (NM_001080441.3, target sequences: TTC36-RNAi-1, 5'-GGAAGAACGAGAAGAAGAT-3', TTC36 RNAi-2, 5'-CGAGAAGAAGATGAAGTTT-3', TTC36-RNAi-3, 5'-GGGCTTCAGCCTACAACAA-3') and a negative control shRNA were designed to construct TTC36 interference lentivirus, and the sequences was inserted into Agell/Ecol I restriction sites of a pLKO.1-Puro vector (Addgene, Cambridge, MA, USA). The coding DNA sequence region of TTC36 synthesized by Genewiz Company (Shanghai, China), was inserted into EcoR I/BamH I restriction sites of a pLVX-Puro vector (Clontech, Palo Alto, CA, USA) to construct the overexpression plasmids. The integrity of the respective plasmid constructs was confirmed by DNA sequencing (Majorbio, Shanghai). Lentiviral particles were generated in 293T cells by co-transfection of lentiviral transfer plasmid pLKO.1-Puro-TTC36 RNAi, pLVX-Puro-TTC36 or control plasmid using Lipofectamine 2000 (Invitrogen), along with the packaging plasmids psPAX2 and pMD2.G (Addgen, USA). After 48 h, the lentiviral supernatants were collected and filtered (0.45-μm size filter, Millipore, Billerica, MA, USA). AGS and BGC823 cells were infected with overexpressed viral supernatants or RNAis. Then, TTC36 expression was evaluated by quantitative PCR (qPCR) and western blotting. TTC36 stable overexpression (oeTTC36) and knockdown (TTC36 RNAi) cell lines were used for further experiments.

### Cell proliferation

Cell proliferation rates were analyzed by the cell counting kit-8 (CCK8) assay. Cell suspension were plated in 96-well plates (3,000 cells/100 μL/well) in quadruplicate and evaluated following a period of incubation (0, 12, 24, 48 and 72 h). After removing the medium, 100 μL 10% CCK-8 (C0039, Bayotime) was added to each well and incubated for an additional 1 h at 37 °C. The absorbance of each well at 450nm was measured in a microplate reader (Perlong, Beijing).

### Cell cycle analysis

The cells were digested with 0.25% trypsin and washed twice with cold PBS, then fixed in ice-cold 70% ethanol and stored at 4 °C overnight. After fixation, the cells were washed in PBS again and centrifuged for 5 min at 1,500 rpm. The cells were incubated with 10 μL RNase enzyme (R8020-25, solarbio, Beijing) at 37 °C for 5 min, and then stained with propidium iodide (PI) to a final concentration of 50 μg/mL in dark for 30 min. Flow cytometric analysis was performed using a BD Accuri C6 instrument (BD Biosciences, CA, USA), and the results were analyzed using FlowJo7.6.1 software (FlowJo LLC).

### Apoptosis assays

Apoptosis cells were quantified with the Annexin V-FITC/PI apoptosis detection kit (C1062, Beyotime, Beijing) according to the manufacturer's protocol. Ninety-six hours post-transfection, cells, including floating cells, were harvested, washed twice with cold PBS and resuspended in binding buffer (10 mM HEPES/NaOH, 140 mM NaCl, 2 mM MgCl). Next, 5 μL of Annexin V-FITC and 5 μL of PI were added in each cell samples and incubated in the dark for 15 min at RT. Positively stained cells were quantified and analyzed with an Accuri C6 Flow Cytometer (BD Bioscience).

### XAV939 treatment

For rescue assay, TTC36 RNAi GC cells and their controls were planted in culture wells and cultured at 37 °C overnight, followed by treatment with or without β-catenin pathway inhibitor XAV939 (Selleck, S1180; 10 μM) for 24 hrs, and subjected to further experiments afterwards.

### Xenograft model assay

Six-week-old male BALB/c nude mice (Shanghai Slack Laboratory Animal Co., Ltd.) for tumor xenograft assays. All animal experiments were subject to approval by the Committee on Ethics of Animal Experiments of the Northwest Minzu University. Twelve nude mice were randomly allocated into two groups (n = 6 mice/group) for treatment with different cells: oeTTC36 AGS cells and vector cells. Before injection, cell viability was assessed by Trypan blue staining. To compare the tumor forming capacity of cells with or without TTC36 overexpression, 5 × 10^6^ of cells were suspended in 0.2 mL PBS, and injected under the right armpit of each mouse. Tumor growth was monitored every 3 days after palpable tumor formation. Tumor volume was measured with a caliper and calculated using the formula L × l^2^ × 0.5, where “L” indicates the large diameter and “l” indicates the small diameter. After the animals were sacrificed, a proportion of tumors were dissected and rinsed twice in normal saline then fixed in 4% neutral buffered formalin. Hematoxylin-eosin (H&E) staining was performed on 5-μm paraffin-embedded sections following standard protocols. Total RNA and protein were isolated from the remaining xenograft tissues respectively, and the expression of TTC36 in each tumor was detected by western blotting and qPCR according to the methods mentioned before. The animal experiments were carried out in strict and minimize suffering of animals, according to recommendations in the Guide for the Care and Use of Laboratory Animals of the Northwest Minzu University.

### Statistical analysis

All data are presented as the mean ± SD. Survival curves were generated by the Kaplan-Meier method. The log-rank test was used to estimate the statistical differences between survival curves. Statistical significance for comparisons between two groups of the gene expression, cell cycle ratio and cell apoptosis levels was determined using the Student's t-test. The comparisons among multiple groups were made with one-way ANOVA. *P* < 0.05 (*P < 0.05, **P<0.01, ***P<0.001) was considered to represent statistical significance. Each experiment was performed in triplicate.

## Results

### Downregulation of TTC36 is associated with poor prognosis of GC patients

**Figure 1:**
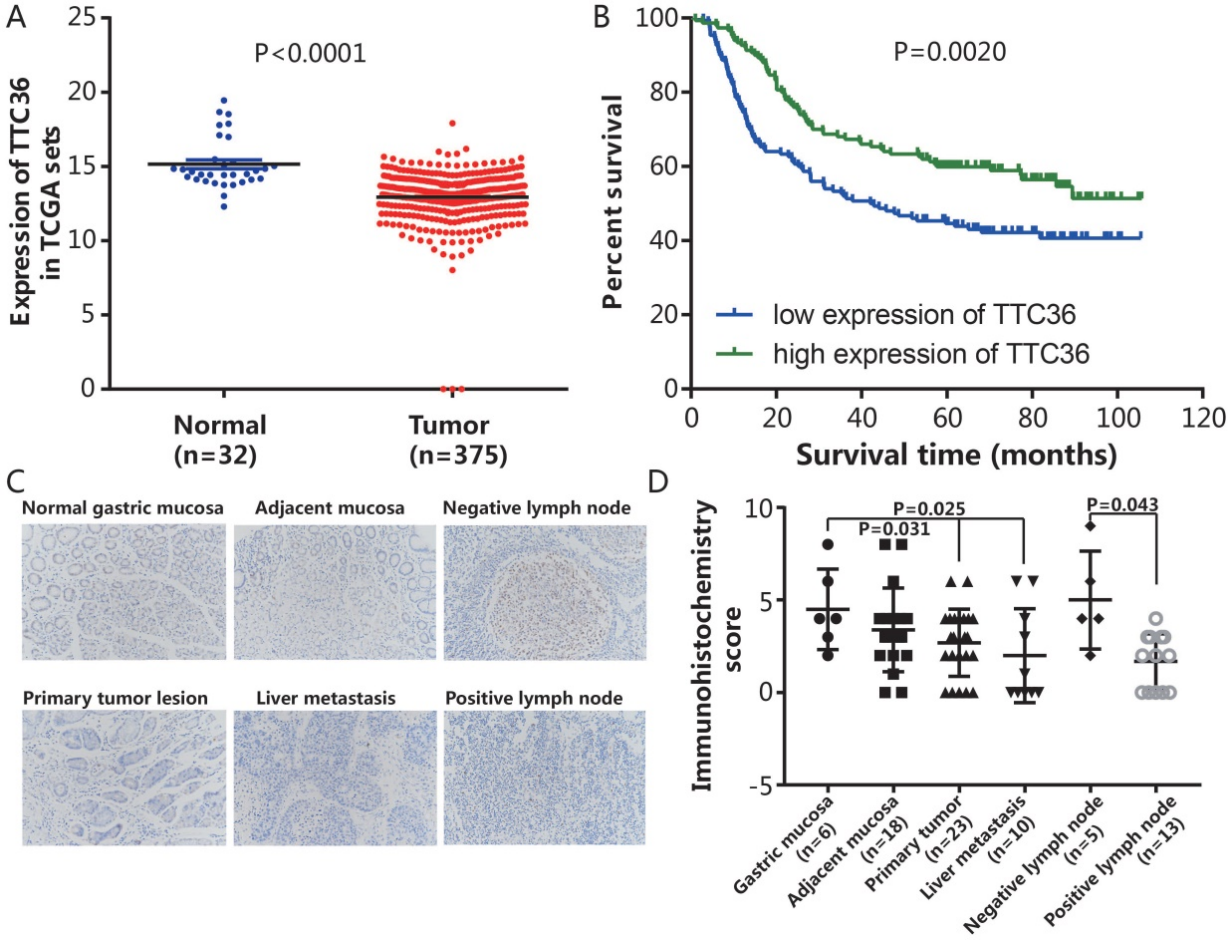
Expression of TTC36 in GC tissues and correlation between patient survivals with TTC36 expression panel. (A) Expression levels of TTC36 in GC (n=375) and normal tissues (n=32) from the TCGA database were analyzed; (B) Survival analysis for all 300 patients with low or high expression levels of TTC36 in GC from the GEO database, by Kaplan-Meier method and Log rank test; (C) A human tissue array containing samples stained for TTC36 from normal gastric mucosa, mucosa adjacent to tumor, non-invasive lymph node, primary tumor lesion, liver metastases and invasive lymph node (×200 magnification); (D) TTC36 expression was high in the non-neoplastic tissues but low in primary tumor, metastasis and invasive lymph node. Significance of differences between groups expressed by *P* values.

To evaluate the promotive or suppressive function of TTC36 expression in GC, the expression of TTC36 was compared between GC and adjacent non-tumor tissues of stomach from TCGA datasets. The results showed that TTC36 mRNA expression was significantly lower in GC tissues compared to normal tissues (Fig. 1A, *P* < 0.0001). To assess the prognostic value of TTC36 expressions in GC patients, survival analyses in cancer were used to analyze the TTC36 expression patterns on the survival of GC patients in a GC specimen expression profile dataset (GSE66229). The results revealed a significant negative correlation between the expression of TTC36 and poor prognoses. The OS of GC patients with low TTC36 expression was markedly worse than those of patients with high TTC36 expression (Fig. 1B, P = 0.002). A total of 80 tissues included in a microslide from different tissue of GC patients were detected by IHC staining (Fig. 1C). The results showed that the expression level of TTC36 from malignant tissues, whether they are primary tumor (P = 0.031), liver metastasis (P = 0.025) or tumor lymph node invasion site (P = 0.043), are less than that of corresponding normal tissues (Fig. 1D), these results provide evidence that decreased TTC36 expression accompanied by GC progression and infiltration of surrounding tissues. Taken together, these findings indicated a tumor-suppressive role of TTC36 in GC, and suggest the clinical significance of TTC36 in GC prognosis.

### TTC36 regulates the proliferation, apoptosis and cell cycle of gastric cancer cells

**Figure 2:**
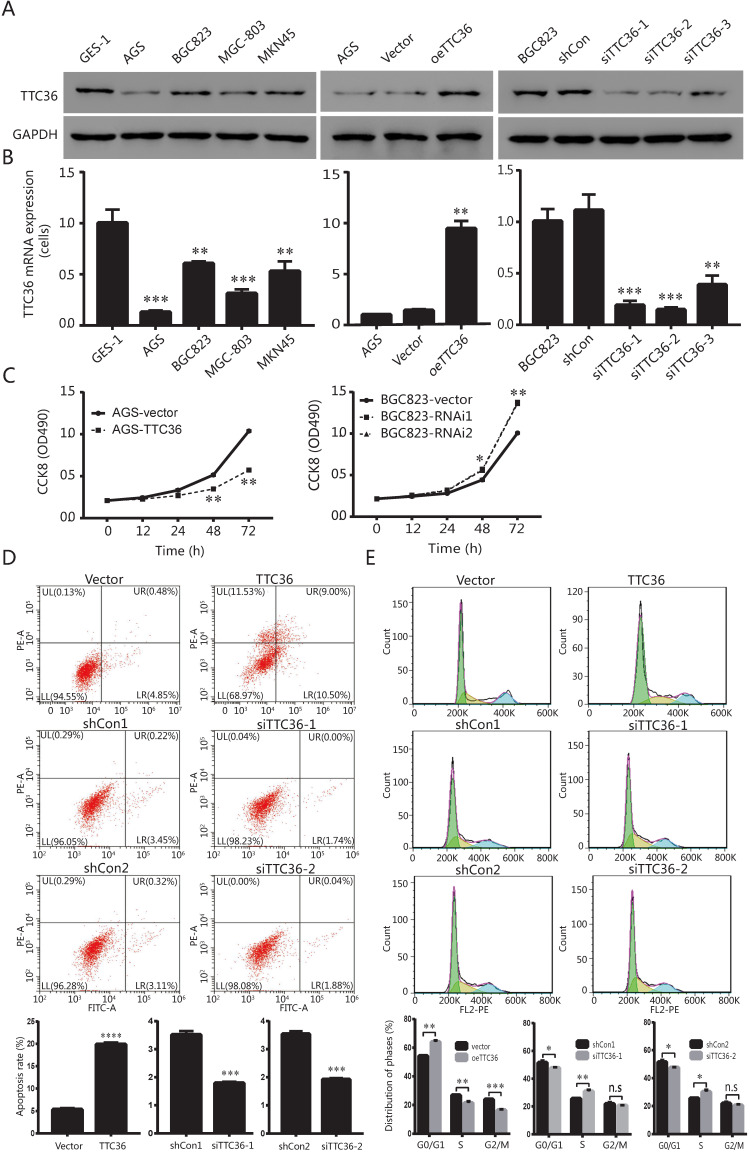
TTC36 is low expressed in human GC cell lines and regulates cell proliferation, apoptosis and cell cycle phases. (A) Protein expression levels of TTC36 in human stomach epithelial cell GES-1 and human GC cell lines (AGS, BGC823, MGC-803 and MKN45). TTC36 was stably overexpressed in AGS cells and silenced in BGC823 cells compared with their control, respectively; (B) Expression levels of TTC36 mRNA in GES-1 and GC cell lines by qRT-PCR. TTC36 overexpressed in AGS and silenced in BGC823 cells were confirmed by qRT-PCR, respectively. GAPDH served as the internal control (compared with gastric mucosal cells, **P < 0.01, ***P < 0.001); (C) Cell viability was analyzed by CCK-8 assay with oeTTC36 AGS and TTC36 RNAi BGC823, respectively; (D) Cell apoptosis rates in oeTTC36 AGS and TTC36 RNAi BGC823 cells; (E) Cell cycle distribution was determined by flow cytometric in oeTTC36 AGS and TTC36 RNAi BGC823 cells. Data were expressed as mean ± SD from three independent experiments. *P < 0.05, **P < 0.01, compared with empty vector group.

In order to investigate whether TTC36 expression is different between normal stomach epithelial cell and GC cells, the mRNA and protein levels of TTC36 were measured in stomach epithelial cell line GES-1 and four gastric carcinoma cell lines, including AGS, BGC823, MGC-803 and MKN45, respectively. As shown in Figure 2A (left) and 2B (left), the expression of TTC36 was dramatically down-regulated in four GC cell lines than that in GES-1 cells. The AGS and BGC823 cell lines were selected for further functional research. AGS cells with relatively low TTC36 expression were used to generate stable TTC36 overexpression cell line by infecting cells with TTC36 overexpressing lentivirus or control virus (Fig. 2A, center). Meanwhile, BGC823 cells with relatively high TTC36 expression were used to generate stable TTC36 knockdown cell line by infecting cells with TTC36 RNAi lentivirus or RNAi-control ones (Fig. 2A, right).

Next, we evaluated the potential role of TTC36 in GC proliferation. CCK8 assays showed that TTC36 overexpression inhibited AGS cell growth, whereas TTC36 knockdown increased BGC823 cell growth compared with the relative control group (Fig. 2C). These data suggest a suppressing effect of TTC36 in GC cell proliferation. It was hypothesized that TTC36 may affect cell proliferation by regulating apoptosis in GC cell lines. We performed flow cytometry assays in TTC36 overexpressed AGS cells and TTC36 knocked down BGC823 cells. Compared with the empty vector group, the percentage of apoptotic cells significantly increased in TTC36 over-expressed AGS cells (*P* < 0.001), and significantly decreased in TTC36 knocked down BGC823 cells (*P* < 0.001) (Fig. 2D). Our results illustrate that TTC36 affects the viability of GC cells in part due to influence cell apoptosis.

Furthermore, we investigated the role of TTC36 in GC cell cycle. Flow cytometry analysis revealed that the proportion of G0/G1 cells in TTC36 overexpressed AGS cells was higher than the control cells. In contrast, knocking down TTC36 in BGC823 cells induced the converse effect (Fig. 2E), and significantly increased the proportion of S-phase cells, which also explained the cell proliferation effect of TTC36 low expression. These results demonstrated that TTC36 overexpression significantly inhibited G1/S transition, whereas, silence of TTC36 led to cell cycle transition from G1 to S phase. These data indicated that TTC36 inhibited cell proliferation by inducing cell cycle arrest at the G0/G1 phase.

### Overexpression of TTC36 inhibits tumor growth of AGS cells *in vivo*

**Figure 3:**
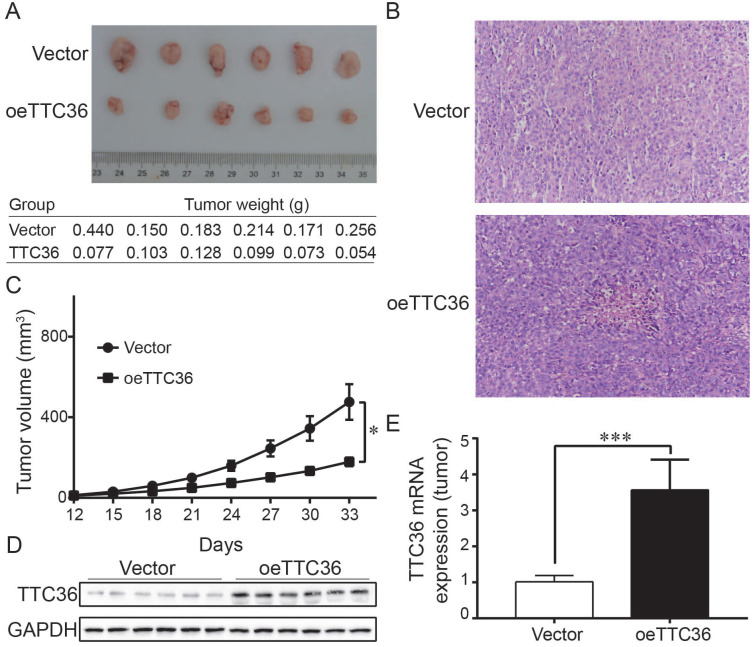
Over-expression of TTC36 inhibits tumor growth and decreases malignancy in nude mice. (A) Comparison of subcutaneously injected xenografts of nude mice; (B) TTC36 decreases malignancy in xenograft tissue. Sections were stained with H&E; (C) Days versus tumor volume curves for the xenografts; (D) Detection of TTC36 protein by Western blotting in xenograft tissue. GAPDH was used as a loading control; (E) qPCR of *TTC36* mRNA expression in xenograft tissue. *GAPDH* served as the internal control (compared with vector group cells). *P < 0.05, ***P < 0.001.

To determine whether TTC36 inhibits tumor growth *in vivo*, a xenograft model was established. Tumor growth was evaluated by measuring the tumor masses and volumes. Tumors derived from TTC36-overexpressing were significantly smaller than those in the vector-expressing group (Fig. 3A). Histological analysis of sections stained with H&E showed that xenografts with high TTC36 expression were more differentiated than those in the vector group (Fig. 3B). Time-volume curves for the xenografts showed that tumors in the oeTTC36 group had a slower growth rate than those in vector-expressing group (*P* < 0.05; Fig. 3C). Western blotting and qPCR analysis showed that expression of TTC36 was appreciable higher in the oeTTC36 group than that in the control group (Fig. 3D, E). In all, these results indicated that TTC36 suppresses the malignancy of GC cells *in vivo*.

### Cell signaling pathway regulated by TTC36

**Figure 4:**
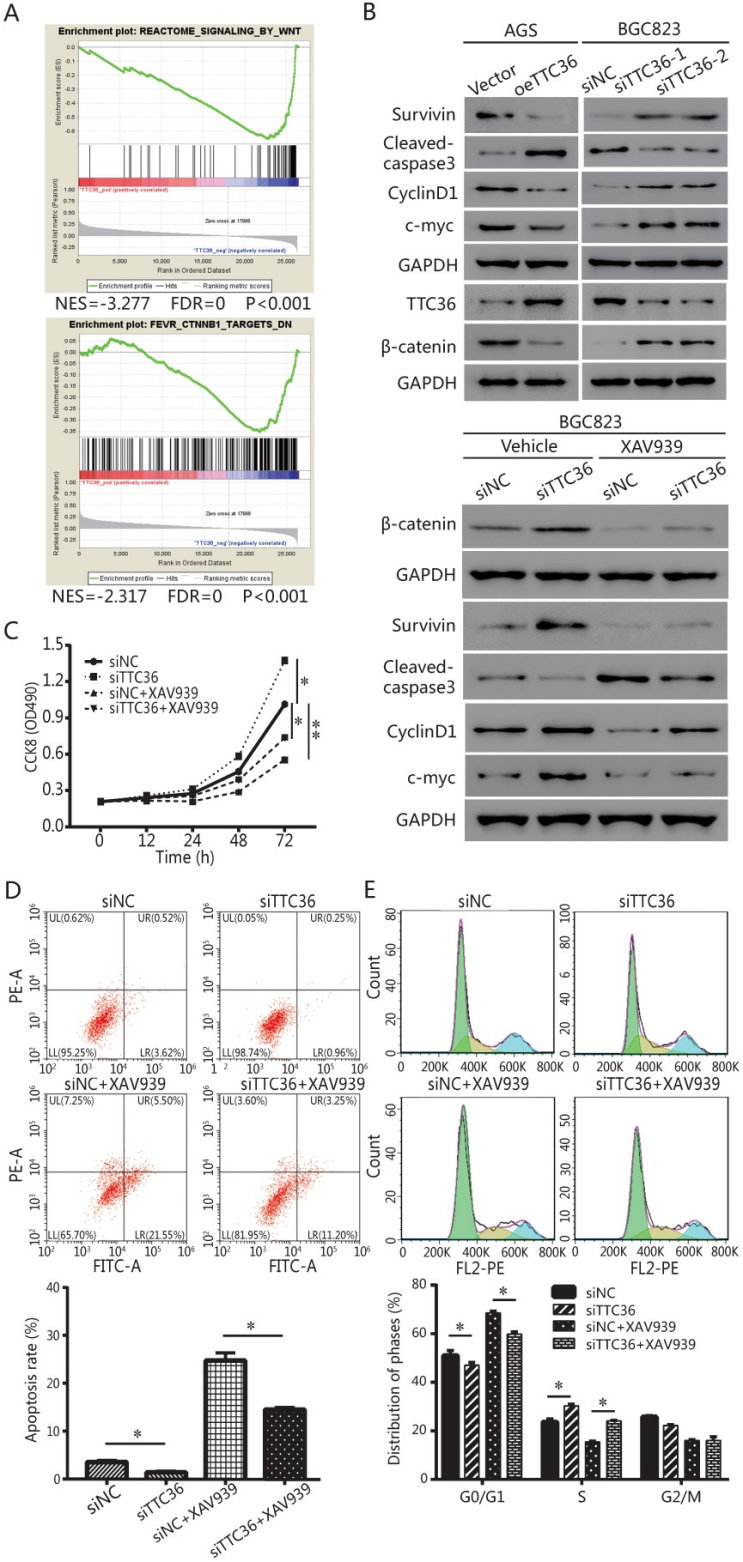
β-catenin signaling pathway was involved in biological functions induced by TTC36 in GC. (A) GSEA analysis showed negative association between low expression of TTC36 and enrichment of the Wnt-β-catenin signaling pathway. The normalized enrichment score (NES) means the degree to which the gene set is overrepresented at the top or bottom of the ranked list of genes; (B) Survivin, cyclin D1, c-myc, cleaved caspase 3 and β-catenin protein expressions in GC cells with or without 10 μM XAV939 treatments. GAPDH was used as a loading control; (C) CCK8 assay for TTC36 RNAi BGC823 cells with or without 10 μM XAV939 treatment; (D) Apoptosis assay for TTC36 RNAi BGC823 cells with or without 10 μM XAV939 treatment; (E) Cell cycle analysis of TTC36 RNAi BGC823 cells with or without 10 μM XAV939 treatment. Data were expressed as mean ± SD from three independent experiments. *P < 0.05.

Gene set enrichment analysis (GSEA) was performed to verify how TTC36 affects the properties of GC cells, and explore pathways that were associated with TTC36 expression in TCGA-STAD cohorts. Enrichment plots of GSEA (Fig. 4A) revealed that TTC36 expression was negatively correlated with enrichment of Wnt and β-catenin gene signature.

The protein levels of TTC36 and β-catenin were detected by western blotting (Fig. 4B). TTC36 expression was strongly negative associated with the β-catenin. Meanwhile, western blotting was used to detect the expression of a series of genes. Cleaved caspase-3 indicates the induction of apoptosis. Cyclin-D1 plays a role as a cell cycle regulatory protein. C-Myc is a key regulator of cellular proliferation and growth factor stimulation. Survivin is a member of the inhibitors of apoptosis protein family. As shown in Figure 4B, Survivin, cyclin D1 and c-Myc expression levels in TTC36 over-expressed AGS cells were lower than those of control cells, the up-regulation of above proteins have been observed in TTC36 silenced BGC823 cells. Cleaved caspase3 were up-regulated in oeTTC36 cells and down-regulated in TTC36 RNAi GC cells. Thus, we currently have evidence that TTC36 regulates cell proliferation, cell cycle and apoptosis by affecting the expression of related genes, and this process may be related to the β-catenin pathway.

To confirm GSEA and western blot analysis of the association between TTC36 and the Wnt/β-catenin pathway, and to study whether the Wnt/β-catenin pathway mediates TTC36 regulating of GC proliferation *in vitro*, a rescue experiment was carried out using TTC36 RNAi BGC823 cells and treated with 10 μM of Wnt/β-catenin inhibitor, XAV939 (Selleck, S1180) for 24 hrs. As shown in Figure 4C-E, XAV939 effectively inhibited β-catenin signaling, and abrogated the effect of shTTC36 on cell proliferation, cell cycle, apoptosis and related gene expressions. Additionally, the protein level alternation of these genes, including Survivin, Cyclin D1, c-Myc and cleaved caspase3, as well as phenotypes of cells were all accordingly rescued in shTTC36 BGC823 cells. Hence, these results proved that TTC36 suppress GC progression via inhibiting Wnt-β-catenin pathway.

### Mechanism of TTC36 affecting β-catenin signaling pathway

**Figure 5:**
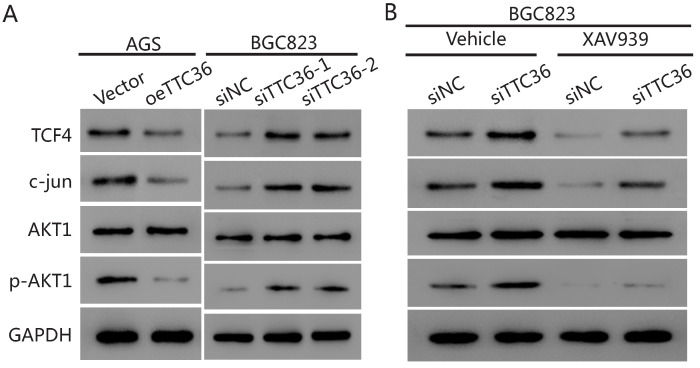
TTC36 regulated TCF4/JUN/AKT pathway is depended on β-catenin in GC cells. (A) Western blot analysis of TCF4, c-jun, p-AKT1 and total AKT1 in oeTTC36 AGS cells and TTC36 RNAi BGC823 cells; (B) Western blot analysis of TCF4, c-jun, p-AKT1 and total AKT1 in TTC36 RNAi BGC823 cells with or without 10 µM XAV939 treatment. GAPDH was uses as a loading control.

**Figure 6:**
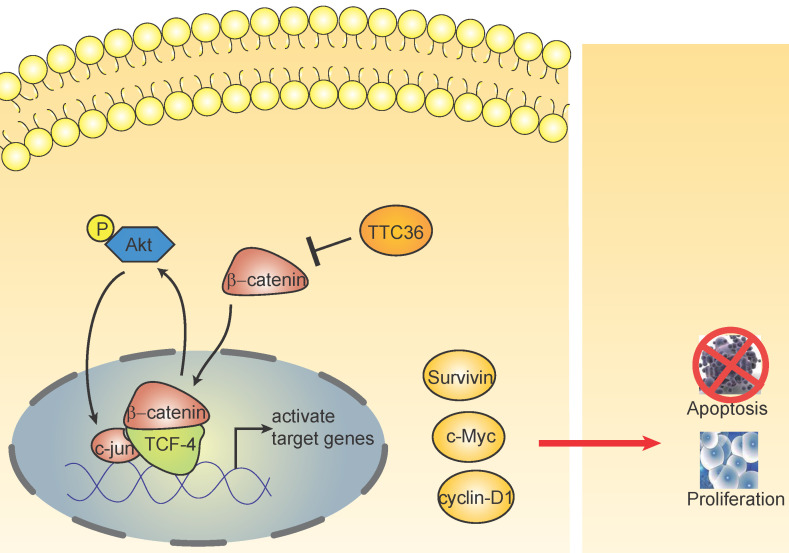
The schematic diagram shows the effect of TTC36 on the feedback loop between β-catenin/TCF4 and Akt signaling cascades in GC.

To further elucidate the underlying mechanism of TTC36 in GC cells, we compared the expression of several cell proliferation associated genes downstream of β-catenin by western blot analysis. The results presented in Figure 5A show that the protein level of β-catenin pathway-related factors (TCF4, c-jun and pAKT1) were also decreased in oeTTC36 AGS cells. On the contrary, knockdown of TTC36 increases the expression of TCF4, c-jun and phosphorylation of AKT in shTTC36 BGC823 cells. XAV939 application rescued expression of TCF4, c-jun and pAKT in shTTC36 BGC823 cells (Fig. 5B). These results further confirm that TTC36 reduces cell proliferation via Wnt-β-catenin and the subsequent downstream signaling events (Fig. 6).

## Discussion

GC is one of the most common malignancies in people worldwide. Although advances in surgical treatment and tyrosine kinase inhibitor therapy have improved disease prognosis, however, disease recurrences still pose a challenge to physicians and patients. With the precision medicine developed rapidly, significant advances in understanding the GC process from both biology and genome have brought target-oriented therapy for advanced GC into clinical and research 14. Although the TCGA project performed comprehensive molecular characterization of gastric adenocarcinoma, the specific molecular mode of action involved in the progression of GC remains still complex and elusive. Therefore, finding and understanding clues about the molecular mode of action are critical for the diagnosis and prognosis of GC.

In the present study, TCGA database and GEO data set revealed that TTC36 was low expressed in GC tissues versus normal tissues and low expression of TTC36 was associated with significantly poor OS rate of patients. The decrease of TTC36 expression accompanied by GC progression and infiltration of surrounding tissues was acceded confirmed by TMA (Fig. 1). Low expression of TTC36 was also confirmed in our GC cell lines. The results revealed that overexpression of TTC36 significantly inhibited cell growth, cell cycle, and enhance apoptosis rate *in vitro*. Opposite effect was confirmed after knocking down TTC36 in BGC823 cell line (Fig. 2). In agreement with our results, Jiang *et al.* 12 reported that TTC36 is dramatically downregulated caused by allele loss and promoter methylation lead to progression of hepatocellular carcinoma. Functional study found that TTC36 could promote cell apoptosis and inhibit tumorigenesis *in vitro* and *in vivo*, as well as chemotherapeutic resistance. In another study, TTC36 was been demonstrated elevated in high grade breast cancer and proliferative vitreoretinopathy (PVR) proliferative membrane, and acted as an oncogene role in regulating cancer biology processes 15. The combination of TTC36 and Hsp70 may contribute to rescue tumor cells from programmed cell death. These divergent studies suggest that TTC36 probably plays different functions in different types of tumors. Our study also provided evidence that TTC36 played the key role in GC progression via inactive of the expression of specific genes such as survivin, cyclin D1 and c-Myc which may stimulate cell proliferation and anti- apoptosis, reduce tumor growth and cell cycle transition and facilitate cell apoptosis 16, 17. More importantly, the significantly inhibition of tumorigenesis has been observed in a xenograft model of TTC36 over-expression GC cells (Fig. 3). This study is based on the clinical significance and functional role of TTC36 in the development of GC and has never been reported before.

Role of Wnt/β-catenin signaling pathway in regulation of cell proliferation and participates in mediating cancer development has been widely accepted 18. The tumors investigated, β-catenin activation has been demonstrated to be essential for tumor cell growth and progression 19-21. For instance, SOX2 enhanced cell viability and invasiveness via targeting the Wnt/β-catenin signaling pathway in renal cell carcinoma 22. HEG1 silencing results in the inactivation of the Wnt/β-catenin signaling pathway and suppressing cancer progression, tumor growth, and angiogenesis in hepatocellular carcinoma 23. Chen *et al.* 24 proved stabilization of Wnt/β-catenin involving in multi-drug resistance in endometrial cancer and revealed the significant and positive correlations of MRP4 with β-catenin and Wnt/β-catenin targets genes in the receptive endometrium by analysis of human endometrial biopsy samples. Studies have shown that the Wnt/β-catenin signaling pathway activated in most of gastric cancers 25, and play an important role in the onset and progress of GC 26. In the present study, TTC36 was found to inactive Wnt/β-catenin signaling pathway and inhibition of Wnt/β-catenin pathway abrogated the effects of TTC36 knockdown on proliferation, cell cycle transition and cell apoptosis (Fig. 4).

Beta-catenin and Tcf-4(T-cell factor 4) are the pivotal components of the canonical Wnt/β-catenin pathway. The β-catenin binds to TCF in the nucleus and induces transcription of Wnt downstream genes 27. Wnt/β-catenin/Tcf signaling pathway have been reported playing crucial roles in the development of many cancers 28-30. This complex regulates the transcription of multiple genes involved in cellular proliferation, survival, and apoptosis, such as c-Myc, Cyclin D and survivin 31. It has been widely reported Akt is regulated by β-catenin/Tcf at the transcriptional level, leading to a positive feedback loop between β-catenin/Tcf signaling and Akt signaling 32-34. In this study, we also found TTC36 may also serve as a medium between Wnt/β-catenin and Akt signaling cascades (Fig. 5). TTC36 attenuate β-catenin/TCF4 pathway plays a vital role in regulating cell growth, cell cycle process and apoptosis of GC cells by modulating AKT1/c-jun expression. These findings suggest that involvement of the Wnt/β-catenin pathway and its target genes in the function of TTC36 in GC (Fig. 6). Thus, we conclude that inactivation of TTC36 might promote GC cell proliferation, at least in part, through activating Wnt/β-catenin signaling pathway.

## Conclusion

In summary, this research revealed that TTC36 is a putative tumor suppresser in gastric cancer. TTC36 extorts a crucial role in the proliferation of GC *in vitro* by activating the β-catenin signaling pathway. The result supports TTC36 as an inhibitor of Wnt/β-catenin signaling pathway that will be beneficial for intervention of GC.
